# Uric Acid and Oxidative Stress—Relationship with Cardiovascular, Metabolic, and Renal Impairment

**DOI:** 10.3390/ijms23063188

**Published:** 2022-03-16

**Authors:** Mihai-Emil Gherghina, Ileana Peride, Mirela Tiglis, Tiberiu Paul Neagu, Andrei Niculae, Ionel Alexandru Checherita

**Affiliations:** 1 Department of Nephrology, “Carol Davila” University of Medicine and Pharmacy Bucharest, 020021 Bucharest, Romania; gherghina_mihai92@yahoo.com (M.-E.G.); al.checherita@gmail.com (I.A.C.); 2 Department of Anesthesiology and Intensive Care, “Carol Davila” University of Medicine and Pharmacy Bucharest, 020021 Bucharest, Romania; mirelatiglis@gmail.com; 3 Department of Plastic Surgery and Reconstructive Microsurgery, “Carol Davila” University of Medicine and Pharmacy Bucharest, 020021 Bucharest, Romania; dr.neagupaul@gmail.com

**Keywords:** uric acid, cardiovascular risk, oxidative stress, chronic kidney disease, outcome

## Abstract

Background: The connection between uric acid (UA) and renal impairment is well known due to the urate capacity to precipitate within the tubules or extra-renal system. Emerging studies allege a new hypothesis concerning UA and renal impairment involving a pro-inflammatory status, endothelial dysfunction, and excessive activation of renin–angiotensin–aldosterone system (RAAS). Additionally, hyperuricemia associated with oxidative stress is incriminated in DNA damage, oxidations, inflammatory cytokine production, and even cell apoptosis. There is also increasing evidence regarding the association of hyperuricemia with chronic kidney disease (CKD), cardiovascular disease, and metabolic syndrome or diabetes mellitus. Conclusions: Important aspects need to be clarified regarding hyperuricemia predisposition to oxidative stress and its effects in order to initiate the proper treatment to determine the optimal maintenance of UA level, improving patients’ long-term prognosis and their quality of life.

## 1. Introduction

Excretion of nitrogenous waste from the body can manifest in three forms: urea, ammonia, and uric acid [1]. Uric acid (UA) is the final product of purine metabolism (adenine and guanine degradation), mostly derived from endogenous synthesis and only a minor part from exogenous sources [2]. Neel’s hypothesis sustains that our ancestors underwent genetic mutations that silenced the gene involved in vitamin C synthesis and UA degradation. It seems that ascorbic acid is a competitor in tubular reabsorption with UA [3]. Its role in kidney stones is well known and is the most obvious link with CKD [4], probably due to urate capacity to precipitate within the tubules or the extra-renal system, especially in obese subjects [5]. Emerging studies support association of high UA levels with onset and increase progression of CKD, cardiovascular risk [6], hypertension [7,8], diabetes mellitus, metabolic syndrome [9,10], and cognitive decline [11]. This time, the pathogenesis is related to pro-inflammatory and pro-oxidative effects of UA. Other current pathogenesis incriminated in the onset and progression of already mentioned diseases are epithelial-to-mesenchymal transition in renal tubular cells, renal vasoconstriction mediated by endothelial dysfunction, and activation of renin-angiotensin system (RAAS) [12]. Some studies reported that high levels of uric acid may exert protective effects in neurological disorders, such as Parkinson’s disease [13], multiple sclerosis [14,15], Alzheimer’s disease [16], and vascular-disease-related dementia [2] through its extracellular anti-oxidative role. In contrast, some researchers sustain the hypothesis that deterioration of kidney function associated with hyperuricemia might be due to co-existing conditions, such as vascular calcifications, hypertension, and obesity [17]. Serum UA has a biphasic role; therefore, many of the studies have reported a U-shaped association with cardiovascular mortality [12].

## 2. Uric Acid Synthesis

As previously mentioned, UA, a ubiquitous product in different life forms [18], can be linked to the development and progression of CKD through various pathogenesis. UA presents a heterocyclic organic structure, C5H4N4O3, with the following characteristics [18,19]:

- White crystals or powder aspect,
- A molar weight of 168.11 g/mol,
- A heavy atom count of 12,
- A melting point >300 °C,
- A water solubility of 60 mg/L (at a temperature of 20 °C).

UA can be produced in/by different organs and tissues, such as liver, intestines, muscles, kidneys, vascular endothelium, and even apoptotic cells, as it is acknowledged that the nucleic acids, adenine, and guanine of these cells are finally degraded into UA [20].

The complex chemical reactions involved in the UA synthesis include two possible pathways—the degradation of adenine and guanine, respectively [18,20,21]:

- Adenine pathway:
  ○ Adenosine monophosphate (AMP) is converted by nucleotidase into adenosine, which is further converted by purine nucleoside phosphorylase into adenine, which, through deamination, is degraded to hypoxanthine.
  ○ AMP can also present a deamination reaction, being converted into inosine monophosphate (IMP), which is converted by nucleotidase into inosine, which is further degraded by purine nucleoside phosphorylase into hypoxanthine.
  ○ The resulting hypoxanthine, under the action of xanthine oxidase, is converted to xanthine.
- *Guanine pathway*:
  ○ Guanosine monophosphate (GMP) is converted by nucleotidase into guanosine, which is further converted by purine nucleoside phosphorylase into guanine, which, through deamination, is degraded to xanthine.
  ○ GMP can also present a deamination reaction, being converted into xanthosine monophosphate (XMP), which is converted by nucleotidase into xanthosine, which is further degraded by purine nucleoside phosphorylase into xanthine.
- The resulting xanthine, through adenine and guanine pathway, under the action of xanthine oxidase, is oxidized to uric acid, which in the normal human body’s physiological conditions, exists as urate, with the following normal range of levels, which are different for men and women: 2.5–7.0 mg/dL in male gender and 1.5–6.0 mg/dL in female gender, respectively. Furthermore, the urate is easily transformed to allantoic acid and ammonia, allowing its renal excretion (almost 200–300 mg/day).

**Figure 1:**
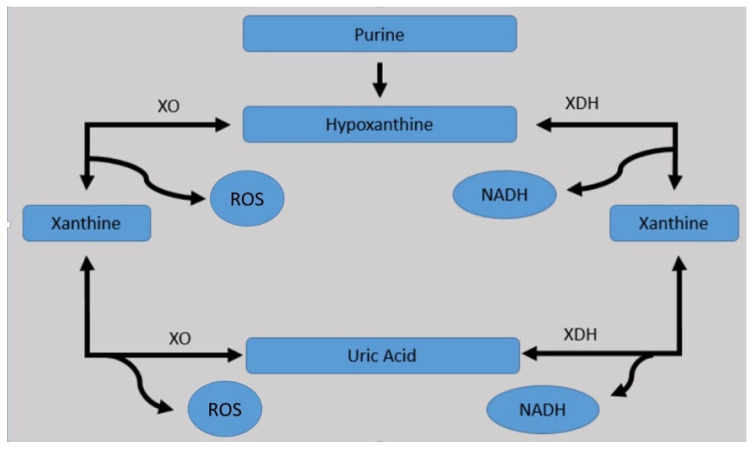
Metabolism of purine through xanthine oxidoreductases. XO, xanthine oxidase; XDH, xanthine dehydrogenase; ROS, reactive oxygen species; NADH, nicotinamide adenine dinucleotide (Modified after: [23]).

Summarizing, there are two pathways for purine generation: (1) the novo synthesis from non-purine compounds (i.e., bicarbonate. amino acids), regulated by phosphoribosyl-pyrophosphate synthetase (PRPP), and (2) the purine salvage, the mechanism controlled by hypoxanthine-guanine phosphoribosyltransferase (HPRT). Purine is then catabolized by xanthine oxidoreductase (XOR), classified in two different isoforms: xanthine dehydrogenase (XDH) and xanthine oxidase (XO). Both of them catalyze the oxidation of hypoxanthine to xanthine and subsequently form UA [3]. XO will be converted in UA and superoxide anion, which will lead to high intracellular amounts of superoxide anions, which may induce cell envelope damage and enhanced mutagenesis. Only mammals have XO type [22]. XDH is the most common form of XOR and, by the conversion through UA, will develop a reduced form of nicotinamide adenine nucleotide (NADH). In conditions that cause exposure to a hypoxic environment, such as advanced cardiac failure, pulmonary disorders, sepsis, intoxications, and others, XDH will be converted into XO [23] (Figure 1). XO has an important role in preventing infections by producing mitochondrial ROS although excess formation will imbalance the oxidative and anti-oxidative equilibrium and subsequently promote a pro-inflammatory status [22]. Furthermore, once UA is synthetized, it will be transported to the circulatory system and excreted through renal and gastrointestinal pathways [24].

Serum UA homeostasis is determined by its production, exogenous contribution, and the balance between renal and intestinal reabsorption and secretion. Hyperuricemia is defined as a UA concentration higher than 7 mg/dL in men, 6 mg/dL in women, and 5.5 mg/dL in children and teenagers [24]. Causes of hyperuricemia are rich purine diets, congenital disorders, tumor lysis syndrome, seizures, rhabdomyolysis [25], hypercatabolic states [26], or drugs (i.e., acid acetylsalicylic, theophylline, mycophenolate, beta- and alfa- adrenergic antagonists, angiotensin-converting enzyme, cyclosporine) [27]. Low serum level of UA may be encountered in large volumes of parenteral fluids, psychogenic polydipsia, inappropriate antidiuretic hormone (SIADH), or in some hepatic diseases (i.e., cholangiocarcinoma, viral hepatitis, primary cirrhosis), immunosuppression, or neoplasia. Hypouricemia can be defined when UA is under 2.0 mg/dL [12].

## 3. Uric Acid Rich Diets

Historically, during the last 100 years, there is a progressive rise in UA serum level, especially in Western diets. Most likely the cause is increased consumption of fructose-containing sugars, a product used more and more because of its high capacity of sweetening [28]. Fructose metabolism is located in the liver, has no negative feedback, and needs a great deal of ATP (adenosine triphosphate) and intracellular phosphate consumption. Through phosphorylation, fructokinase converts fructose to fructose 1-phosphate (fructose 1-p). Consequently, fructose 1-p aldolase (or aldolase B) converts fructose 1-p to dihydroxyacetone phosphate (DHAP) and D-glyceraldehyde. Because these reactions need ATP consumption, intracellular phosphate decreases. This will stimulate AMP deaminase (AMPD), which will induce AMP degradation to IMP (inosine monophosphate), increasing the rate of purine degradation and UA synthesis [3]. A meta-analysis from 2018 found seven types of food (beer, wine, liquor, soft drinks, poultry, potatoes) and meat (i.e., beef, pork, lamb) that were associated with high UA serum level and another eight foods with reduced serum UA level but with high protein levels (i.e., eggs, skim milk, brown bread, non-citrus fruits, cheese, cereals, etc.) [29]. Some studies confirm that high-purine vegetables were not associated with hyperuricemia or gout involvement but should be avoided in patients with advanced kidney disease or gout [2].

## 4. Possible Genetic Disorders Involved in Uric Acid Over-Synthesis

Lesch–Nyhan syndrome is an inherited disorder caused by hypoxanthine-guanine phosphoribosyl transferase (HPRT) deficiency that can be associated with uric acid overproduction and gout. The HPRT is one of the enzymes responsible for “recycling” purine nucleotides. Another genetic disorder associated with hyperuricemia and gout is over-expression of phosphoribosyl pyrophosphate synthetase (PRPP). This enzyme contributes to UA formation due to its role in purine de novo synthesis starting from ribose-5 phosphate and ATP. Glucose high-flux metabolism can also lead to hyperuricemia by stimulating pentose phosphate pathway and generating excessive amounts of ribose-5 phosphate [30].

## 5. Uric Acid Excretion

Considering that above the normal range values, especially for levels > 6 mg/dL in both genders, UA can be precipitated into urate crystals, which can lead to the onset and progression of various diseases (i.e., gout), it is important to underline the process of UA elimination and regulation [18,31,32]:

- Most of the urate is excreted by the kidneys (only one-third is gastro-intestinally eliminated), which involves three phases: filtration, reabsorption, and secretion; knowing that it is not bound to the proteins, it is easily ultra-filtrated by the glomeruli. In the proximal renal tubules, most of it is reabsorbed (~95–98%), and then, secretion occurs, with 10% of filtered uric acid being further excreted by the kidneys [33].
- These processes of urate elimination and regulation are influenced by different proteins/transporters:
  ○ URAT1 (urate transporter 1)—it belongs to the organic anion transporter (OAT) family and is a urate exchanger in the apical region of the proximal renal tubules, being encoded by SLC22A12 (solute carrier family 22, organic anion/urate transporter, member 12) gene. It is responsible for the urate reabsorption, therefore having an important key role in maintaining serum UA normal values.
  ○ BCRP (breast-cancer-resistance protein)—a transporter encoded by ABCG2 (adenosine triphosphate binding cassette subfamily G member 2) gene that recently was observed to have an essential contribution in urate excretion. In CKD, high urate levels induce a more active intestinal transporter of UA through ABCG2 increased expression and/or functionality [34].
  ○ GLUT9 (glucose transporter 9)—a transporter for urate, with similar roles as URAT1, located in the basolateral region of the proximal renal tubules.
  ○ SGLT2 (sodium-glucose transporter 2)—recent data suggested its contribution in transporting urate and consequent involvement in its excretion.

**Figure 2:**
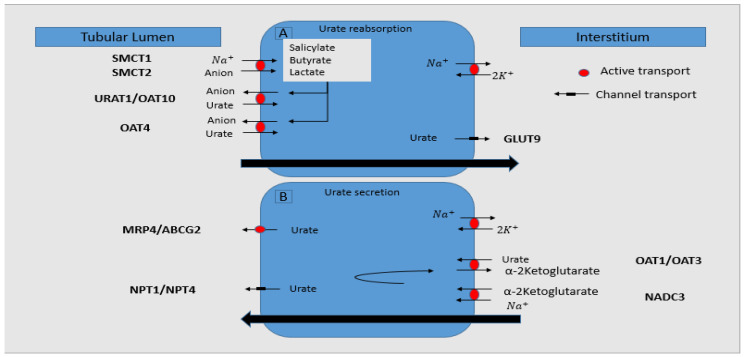
(**A**) SMCT1 and SMCT2 reabsorb Na-dependent anions and raise intra-cellular concentration. URAT1/OAT10/OAT4 exchange intracellular anions with tubular urate, which will exit the cell via GLUT9. (**B**) Na and alpha-ketoglutarate will enter the cell through NADC3. Basolateral OAT1/OAT3 exchanges plasma urate with intracellular alpha-ketoglutarate. Intracellular urate exit is accomplished through voltage channels NPT1/NPT4 or ATP-driven MRP4. SMCT1,sodium-monocarboxylate co-transporter 1; SMCT2, sodium-monocarboxylate co-transporter 2; Na^+^, sodium; K^+^, potassium; URAT1, urate transporter 1; OAT10, organic anion transporter 10; OAT4, organic anion transporter 4; OAT1, organic anion transporter 1; OAT3, organic anion transporter 3; MRP4, multidrug resistance protein 4; ABCG2, adenosine triphosphate binding cassette subfamily G member 2; NPT1, Na-phosphate transporter 1; NPT4, Na-phosphate transporter 4; GLUT9, glucose transporter 9; NADC3, sodium-dependent dicarboxylate cotransporter 3 (Modified after [35]).

In fact, to be accomplished, urate reabsorption (Figure 2) needs two types of apical transporters [35]. One of them is responsible for sodium-anions cotransport (sodium-monocarboxylate co-transporters SMCT1 and SMCT2) from tubular space into the cell, and the other type (URAT1, OAT10, and OAT4) exchanges intracellular anions with luminal urate. High concentration of anions, such as lactic acid [36], salicylate, nicotinate [37], pyrazinamide [38], butyrate, and acetoacetate [39], increase urate reabsorption and induce hyperuricemia. As already mentioned, URAT1 is the main anion–urate exchanger; therefore, many drugs, such as Probenecid, Benzpromarone, Fenofibrat [40], or Losartan [41], that inhibit URAT1 lead to hypouricemia. From intracellular space, urate will be transported to circulation via GLUT9, encoded by SLC2A9 (solute carrier family 2, organic anion/urate transporter, member 9) gene. Inherited GLUT9 deficiency prevents reabsorption with great amounts of UA loss in urine, approximately 150% of filtered urate, with a proof for secretion mechanism being predominant in the absence of reabsorption [42].

Urate secretion (Figure 2) is made by basolateral exchanger alfa-Ketoglutarate-urate named OAT1 and OAT3, through which serum urate is driven to intracellular space in exchange with alfa-ketoglutarate. Intracellular urate will exit the cell through the apical pole, mediated by a cell negative membrane potential named NPT1 and NPT4 (Na-phosphate transporter) and by MRP4 channel (multidrug resistance protein 4), an apical ATP- driven efflux pump encoded by ABCG2 gene [35,43]. The MRP4 channel is stimulated by xanthine-oxidase inhibitors, such as allopurinol and its metabolite oxipurinol [44]. Loop diuretics inhibit NPT4, suggesting a role in reduced secretion of urate in hyperuricemia-associated diuretics syndrome [45].

Other control factors in the balance of serum UA can be volume status (enhances reabsorption) [46,47], low-salt diet (enhances reabsorption) [48,49], insulin [50,51,52], angiotensin II [53], epinephrine, and PTH, which raises serum UA through unknown mechanisms [54]. In addition, an important factor in controlling serum UA is oxidative stress, which modulates production, excretion, and reabsorption of urate [55].

## 6. Pathogenic Potential of Serum Uric Acid in Renal Impairment

There are several mechanisms incriminated in the association of renal impairment and hyperuricemia. First of all, the most popular mechanism is represented by monosodium urate (MSU) crystal depositions in the joints, kidney, and other tissues. Studies showed that over 6.5 mg/dL, serum becomes supersaturated in MSU. Other pathogenic mechanisms that correlate uric acid with kidney disease are intracellular pro-oxidative properties, induced endothelial dysfunction, induced renal fibrosis, and induced glomerulosclerosis [25]. According to emerging studies, UA stimulates transcription of growing factors, nuclear factor kappa-light-chain-enhancer of activated B cells (NF-kB), and various vasoactive substances, such as endothelin, angiotensin II, or thromboxane. Additionally, UA is implicated in decreased oxide nitric (ON) synthesis, inhibiting endothelial cell proliferation and migration [35]. Taken together, all these effects provide strong correlation between UA and arterial stiffening from CKD [56]. Through RAAS, UA stimulates xanthine oxidase, NADPH oxidase [57,58], and proliferation of smooth muscle cell [59,60]. Renal fibrosis represented by tubulointerstitial fibrosis and glomerulosclerosis was proven through a clinical study of 1700 biopsy-confirmed patients with hyperuricemia. The incriminated pathways are activation of epithelial- and endothelial-to-mesenchymal transition (EMT, EndoMT), which will activate fibroblasts and myofibroblasts and subsequent renal fibrosis [25]. Other studies associate inflammation markers, such as C-reactive protein (CRP) or TNF-alfa, as directly proportional with serum UA concentrations [61,62]. Some researchers consider that serum UA can be a strong predictor of acute kidney injury without correlation with the baseline renal function [63].

## 7. Bimodal Role of Uric Acid in Oxidative Balance

Biologically, uric acid can have not only pro-oxidative but also anti-oxidative properties. Extracellular UA mainly acts as an anti-oxidant, also named “scavenger”, with certain benefits in neurological diseases [64]. At the extracellular level, UA reacts with superoxides to form allantoin, with peroxynitrite to form triuret [65], or with nitric oxide to form 6-aminouracil, representing examples of anti-oxidative properties [66]. UA is one of the major antioxidant products from humans, being responsible for up to 55% extracellular capacity of free radical antioxidant scavenging [2]. Additionally, extracellular uric acid reacts with myeloperoxidase with generation of hydroperoxide urate, a compound with pro-oxidative properties [67]. In contrast with its extracellular double role, intracellular UA performs only pro-oxidative actions through activating nicotinamide adenine dinucleotide phosphate (NADPH) oxidases [68] but also through other several mechanisms, such as reducing endothelial levels of antioxidant nitric oxide (NO), activating peroxynitrite-mediated oxidation of lipids, or by stimulating pro-inflammatory biomarkers. The same authors considered that oxidative stress (OS) from hyperuricemia may play a role in aging and apoptosis of endothelial cells [17]. NADPH oxidases are involved in ROS production that can lead to pro-inflammatory signaling through mitogen-activated protein kinases (MAPKs). Hyperuricemia associated with oxidative stress is responsible for several pathophysiological responses, such as DNA damage, oxidations, inflammatory cytokine production, and cell apoptosis. The main site of oxidative phosphorylation is in the mitochondria, which produce ROS by transferring electrons from electron transport chain complexes to free oxygen radicals. Apparently, hyperuricemia is responsible of mitochondrial calcium overload, which induces dysfunctionalities in sodium and calcium mitochondrial exchange that will lead to ROS production [25]. The fact that hyperuricemia induces oxidative stress, which stimulates inflammation, raises the possibility in which hyperuricemia might benefit not only from urate-lowering agents but also from agents that target immune system aspects, such as interleukin-1, autophagy, or other pro-inflammatory factors [66].

## 8. Oxidative Stress and Uric Acid

Oxidative stress is a non-traditional risk factor for all causes of mortality observed frequently in CKD. The onset or progression of CKD, hypertension, metabolic syndrome, and insulin resistance are closely related with a pro-oxidative state generated by chronic inflammation. Oxidative stress is a pathological imbalance between pro-oxidative and anti-oxidative factors in favor of pro-oxidants. The main anti-oxidative factors are superoxide dismutase (SOD), glutathione peroxidase, catalase, and NO. According to a study from 2018, the main reactive oxygen species (ROS) generators are NADPH oxidase, xanthine oxidase, mitochondrial enzymes, myeloperoxidase, lipoxygenase, and uncoupled NO synthase. Xanthine oxidase enzymes are found not only in endothelial cells but also in plasma and are considered highly generators of superoxide and hydrogen per-oxide. It stimulates smooth muscle cell proliferation and LOX-1, a lectin receptor that enhances deposition of oxidized LDL, with important roles in atheroma plaque formation. Emerging studies consider that LOX-1 induces endothelial apoptosis, decreases NO production, and reduces expression of scavengers’ receptors from endothelial lesions. Thereby, LOX-1 is seen as one of the main targets in oxidative stress treatment. Oxidative stress markers can be represented by high levels of manoldialdehyde (MDA), peroxinitrite, or advanced glycosylation products (AGEs) and reduced SOD [69].

## 9. Non-Interventional Studies Focused on Uric Acid

Association between hyperuricemia and onset or progression of CKD is a well-known topic, but it is difficult to establish if uric acid induces kidney disease progression or if it can be an independent factor of renal impairment. Strong evidence of hyperuricemia and progression of kidney disease is given by the protein restriction recommended in early-stage CKD, also beneficial in decreasing UA, an end product of protein metabolism [70]. Large studies, such as National Health and Nutrition Examination Survey (NHANES) and German Chronic Kidney Disease (GKKD), support a relationship between hyperuricemia and renal function decline. Additionally, a meta-analysis including 18 prospective studies and 431,000 patients supported that a high level of UA is correlated with higher incidence of onset and accelerating progression of CKD [68]. Two meta-analyses from 2011 and 2015 conducted on 18 prospective cohorts including 55,607 patients [71], respectively, and 25 studies including 97,824 patients [72] support a moderate raise in arterial pressure values correlated with high levels of serum UA. In addition, both of them consider that hyperuricemia is an independent factor of hypertension. Another meta-analysis from 2013 in which were included 32,016 patients from seven studies, support serum UA as an independent factor in onset of diabetes mellitus and onset of metabolic syndrome [73]. In 2008, Hsu et al., found that elevated serum UA was an independent factor for end-stage renal disease in a study on 175,000 subjects after a 25-year follow up [74]. The Modification of Diet in Renal Disease study from 2009 on 838 CKD stage 3–4 patients did not find an association between hyperuricemia and end-stage-renal-disease; instead, the authors sustained that hyperuricemia significantly raises the risk of all-cause and cardiovascular mortality [75]. Luo et al., indicated in a meta-analysis from 2019 on 10 studies (26,660 subjects) that every 1 mL/dL raise in levels of UA was associated with a risk of cardiovascular mortality of 12% in CKD patients [76]. Tsai et al., in a retrospective longitudinal study on 739 subjects, found an increased risk of progression to renal failure with 7% for each 1 mg/dL UA-level increase [77]. In 2018, Zhou et al., supported a directly proportional association between serum UA, IL-6, TNF-alfa, MDA, and indirect proportional with SOD. Thereby, he suggested that hyperuricemia is an independent risk factor in hypertension, myocardial ischemia, and atherosclerosis through its pro-inflammatory and pro-oxidative roles [78]. Additionally, several recent studies suggested an association between CRP and hyperuricemia [79]. The classic murine model was represented by administration of oxonic acid (an uricase inhibitor) on mice with doubling or even tripling serum UA concentration that was associated with higher arterial pressure values through RASS stimulation, oxidative stress induction, and NO reduction. Later effects were represented by hypertension with microvascular changes independent of serum UA levels [35]. A clinical trial from 2004–2008, including 14,267 subjects with type 2 diabetes, metabolic syndrome (50% of total cohort), hyperuricemia (14% of total cohort), and normal renal function, concluded that 14% of the patients developed CKD (eGFR < 60 mL/min), and the incidence of low eGFR was higher in subjects with hyperuricemia and metabolic syndrome during the 4-year follow-up [80].

## 10. Mendelian Randomized Studies Focused on Uric Acid

Mendelian studies and other genetic studies were used to eliminate potential confounders and reverse causality. For genotype, the studies used SLC2A9 gene polymorphism, responsible for 2% of serum UA variability. Oikonen et al., found no association between uric acid and intima-media thickness in 2012 [81]. In a study from 2013, Palmer et al., reported no association between SLC2A9 polymorphism and hypertension or ischemic heart disease [82]. In 2014, Sedaghat et al., also found no association between hyperuricemia induced by 30 genetic polymorphisms and hypertension [83]. In contrast, in 2014, Mallamaci et al., associated hyperuricemia with hypertension due to SLC2A9 polymorphism [84]. In addition, in 2015, Kleber et al., supported the relationship between hyperuricemia resulting from eight genetic variants and cardiovascular mortality [85]. Furthermore, Hughes et al., advanced the idea that there is an association between uric acid genetic score and renal function improvement [86]. It appears that Mendelian randomization studies evidenced contradictory results regarding the relationship between uric acid genetic risk and cardiovascular disease or renal impairment.

## 11. Available Treatment Option for Lowering Uric Acid

Allopurinol is a purine-like XO inhibitor and Febuxostat and Topirostat are non-purine XO inhibitors. XO inhibitors are the first line in the treatment of hyperuricemia. In November 2017, the U.S. Food and Drug Administrations emitted a warning alert related to the use of Febuxostat due to its potential correlation with an increased risk of cardiovascular mortality, and therefore, it must be used with caution [23].

The second-line therapy is attributed to uricosuric agents. Probenecid and Benzpromarone act through inhibition of URAT1 and GLUT9. Recently, it was shown that the use of Benzpromarone can be associated with a lower risk of stroke in patients diagnosed with gout [87]. It should be mentioned that Benzpromarone is considered one of the four drugs linked to severe hepatotoxicity, and for this reason, it was withdrawn in the USA [88].

A new generation of uricosuric has been considered, such as Lesinurad (approved by U.S. Food and Drug Administration in 2015), Arhalofenate, and Dotinurad (developed in Japan in 2018, not yet approved by FDA), which are selective inhibitors of URAT1 and OAT4 [89]. Furthermore, a new generation of XO inhibitor, named 3,4-dihydroxy-5-nitrobenzaldehyde (DHNB), is being studied, with similar properties as Allopurinol but with less toxicity and with direct antioxidant capacity, reducing free radicals and ROS productions [90].

For recombinant and pegylated uricases, other available options for lowering serum uric acid are limited only by parenteral infusion and, presenting an addition, the possibility of producing antidrug antibodies [91].

Inhibitors of SGLT2, a form of treatment for diabetic control through inhibition of reabsorption of glucose, sodium, and uric acid in proximal tube, represents a promising class drug in lowering uric acid, especially in diabetic and CKD subjects. Apparently, SGLT2 inhibitors are associated with a better control of uric acid, improvement of cardiovascular risk, and also slowing the progression of kidney disease [81]. SGLT2-inhibition increases intraluminal glucose, which will interact competitively with uric acid reabsorption via GLUT9b [92]. Currently, there are three oral SGLT2 options approved by the U.S. Food and Drug Administration and European Medicines Agency, recommended for patients with type 2 diabetes mellitus and CKD with eGFR > 30 mL/min/1.72 m^2^. Studies indicated significant improvement in upregulation of angiotensin, reductions of UA, oxidative stress, arterial stiffness, inflammation, and body weight [93]. Other potential advantages from SGLT2 inhibitors may be reduction of anti-inflammatory, anti-oxidant, and anti-fibrotic markers [94]. In addition, studies with Ipragliflozin, Dapagliflozin, and Empagliflozin found decreased levels for oxidative stress and macrophages markers (MCP-1, NF-kB, 8-OhdG, L-fatty acid, interleukin-6, monocyte-attractive-protein-1) [95,96,97]. Regarding adverse effects, the most commonly observed were genital infections, urinary tract infections, bone fractures, or diabetic ketoacidosis [89,98].

Other potential beneficial therapies:

- Estradiol and Losartan are also URAT1 and GLUT9 inhibitors, whereas Fenofibrate acts only through URAT1 inhibition [99]. Despite higher prevalence of CKD in women, a recent study from nationwide Swedish Renal Registry—CKD evidenced that males are prone to a higher eGFR decline. Apparently, more and more studies sustain that sex hormones, especially estrogen, exerts renal and cardiovascular protective proprieties. This may also be a reason for lower levels of uric acid in women [100].
- Vitamin E is a nutrient lipid soluble with pleiotropic benefits in protecting the integrity of cell membranes through ROS scavengers and by blocking the chain of oxidative reactions [101].
- Vitamin C or ascorbic acid and NO are involved in a complex relationship. It has been suggested that ascorbic acid is a potent antioxidant and reduces serum UA levels by increasing urinary excretion, inhibiting AU synthesis, and by directly decreasing ROS-derived cell damage. Flavonoids and other polyphenols due to their anti-oxidative capacity act like superoxide and XO inhibitors, resulting in suppression of ROS and UA synthesis [102].
- ʟ-Arginine and *N*-Acetylcysteine also known as strong antioxidants and have shown repeatedly good results in efficient anti-oxidative properties in CKD. ʟ-Arginine is a substrate for NO synthesis [17].
- An NHANES study in 14,758 subjects sustained that only tea and coffee consumption was associated with low levels of serum UA [103].

## 12. Interventional Studies Focused on Uric Acid

Several studies showed that xanthine oxidase inhibitors mitigate endothelial dysfunction and reduce atherosclerosis risk in smokers [104,105]. Another study proved the beneficial effects of xanthine oxidase inhibitors in hyperuricemia-induced endothelial lesions in contrast with uricosuric treatments. Kang et al., showed in their study, which included 113 patients with CKD stage 3A, the association between oxidative stress and kidney disease progression using xanthine oxidase inhibitors versus placebo [57]. Furthermore, there are studies that supported the benefits of xanthine oxidase inhibitors in slowing kidney disease progression and reducing arterial pressure [106]. A meta-analysis from 2012, which included 10 studies, showed a decrease with 3.3 mmHg of systolic blood pressure and with 1.3 mmHg of diastolic blood pressure in pre-hypertensive patients with UA levels between 5–7.6 mg/dL, treated with xanthine oxidase inhibitors [107]. Another study from 2010 conducted on 113 patients with CKD (estimated glomerular filtration rate—eGFR < 60 mL/min/1.73 m^2^) treated with Allopurinol 100 mg/dL versus continuum initial therapy showed a lower level of CRP (from 4.4 to 3.0 mg/dL) and a rise in eGFR with 1.3 mL/min/1.73 m^2^ versus a decrease of 3.3 mL/2 years [108,109]. In 2014, Bose et al., in a meta-analysis of eight trials on 476 subjects, reported that treatment with Allopurinol, retarded kidney disease progression in five from eight trials [110]. Lee et al., in 144 patients with CKD stage 3 and hyperuricemia, followed from 2005 to 2018 in treatment with Allopurinol vs. Febuxostat, found that subjects on Febuxostat had significantly lower mean serum UA and higher mean eGFR values for 4 years [111]. In 2019, Zhang et al., demonstrated on a cohort of 152 CKD stage 2–3 with hyperuricemia in treatment with Allopurinol vs. Febuxostat that mean eGFR was higher on Febuxostat group after 6 months of follow up [112]. A meta-analysis from 2020 focused on patients diagnosed with CKD stage 5, which included 6000 subjects, compared treatment with Allopurinol vs Febuxostat. The median follow up was 0.72 years, during which significantly fewer patients on Febuxostat started renal replacement treatment [113]. Mauer et al., suggested that UA reduction with Allopurinol slows down the progression of kidney disease in diabetics, and more than that, they considered UA a strong predictor for diabetic nephropathy [114]. Another meta-analysis from 2018 on 12 randomized controlled trials with 832 CKD subjects admitted that lowering UA serum level was associated with significant raise in eGFR, with the mean difference of 3.88 mL/min/1.73 m^2^ between the patients who received uric-acid-lowering therapy and those that did not [115]. In contrast, in 2017, Sampson et al., through a meta-analysis of 12 studies on 1187 subjects, reported that urate-lowering therapies available (Febuxostat, Allopurinol, Probenecid, Sulfinpyrazone, Benzbromarone, Pegloticase, and Rasburicase) showed no benefits. Furthermore, one study evidenced that uric-acid-lowering therapy increased CRP [116]. Su et al., compared placebo vs. acid-lowering agents in a meta-analysis from 2017 in 16 trials that included 1211 subjects and found that those in uric-acid-lowering therapy group presented a 55% relative reduction in the risk of kidney failure events and 60% reduction of cardiovascular events [117]. In 2020, Chen et al., in an overview that included 28 trials involving 6468 subjects, showed that urate-lowering therapy did not reduce the major cardiovascular events, death, or kidney failure [118]. An extensive review, which involved 15 systemic reviews, 144 observational meta-analyses, 31 randomized controlled meta-analysis trials, and 107 Mendelian randomization studies, sustained that the only convincing evidence of a role of serum UA is in gout and nephrolithiasis [119]. Lin et al., in a meta-analysis from 2019 on 11 trials and 1317 subjects, found a significant rise in eGFR for stage 3–4 CKD patients in treatment with Febuxostat [120]. These substantial information regarding UA influence on renal impairment, cardiovascular events, etc., are summarized in Table 1.

### Current Guidelines Recommendations

- In 2020, the Guideline for Management of Gout by the American College of Rheumatology (ACR) recommended initiating urate-lowering therapy for gout patients with more than one subcutaneous tophus, evidence of radiographical damage attributable to gout, or frequent gout flares (>2 per year) [121].
- Japanese guidelines for gout recommended to treat asymptomatic hyperuricemia in patients with UA serum level over 8 mg/dL and complications, such as CKD, urolithiasis, hypertension, cardiovascular disease, diabetes mellitus, and metabolic syndrome, or in patients with UA serum level over 9 mg/dL [122].
- In 2017, EULAR recommendations for gout management suggested Allopurinol as first-line treatment. If target UA level could not be managed (serum UA < 6 mg/dL), the use of Febuxostat or other uricosuric agent was indicated. As third line, a combination of uricosuric and XO inhibitor was suggested. Pegloticase was recommended only for refractory gout [123].

Furthermore, although in Europe, uric acid is not considered an independent risk factor, the majority of interventional studies, even though on small cohorts, prove the benefits of xanthine oxidase inhibitors therapy in asymptomatic hyperuricemia. On the other hand, it is important to acknowledge the risks associated with therapy administration. There were reported rare cases of Stevens endothelial lesions Johnson syndrome, secondary vasculitis, hepatitis, or even acute kidney injury. It is important to remember that UA takes a unique position in uremic retention because its serum concentration can be controlled selectively by specific medication [124]. Some researchers proposed to treat asymptomatic hyperuricemia under the presence of urate crystals in urine sediment and/or by asymptomatic damage of joins identified through musculoskeletal ultrasound. Apparently, these signs appear long term before gout is evident. Musculoskeletal ultrasound reveals asymptomatic urate depositions by hyperechoic enhancement of the cartilage surface, cartilage with double contour, intra-articular hyperechoic clouds, bone erosion, synovitis, or periarticular power signal [125].

## 13. Conclusions

According to the meta-analyses and pilot studies presented, high levels of serum UA are related with a prooxidative and proinflammatory state. Due to the fact that xanthine oxidase inhibitors have more benefits regarding endothelial functions and slowing kidney disease progression in contrast with uricosuric agents and the fact that Mendelian randomization studies evidence conflictual results, we are prone to establish oxidative stress as the pathogenic factor instead of uric acid itself. However, the studies have many con-founding factors and include a small number of patients. Therefore, to establish serum uric acid as an independent risk factor for CKD, hypertension, cardiovascular events, or metabolic syndrome, more studies with larger cohorts are needed. Regardless of whether hyperuricemia is considered or not an independent risk factor for renal impairment, cardiovascular risk, or metabolic syndrome, we know that its formation pathway and also its intracellular reactions contribute to oxidative stress, which is considered as a potential CKD progression factor. Therefore, important questions need to be clarified as to whether hyperuricemia or oxidative stress are responsible for kidney damage in order to initiate the proper treatment, to determine the optimal maintenance of serum UA level, and improvement of patients’ outcome and quality of life.

## Figures and Tables

**Table 1:** Interventional studies focused on uric-acid-lowering treatment in CKD patients with hyperuricemia.

Study	Studied Medication	Included Subjects	Results
Agarwal et al. (2012, meta-analysis) [107]	Allopurinol vs. placebo 1:1	10 clinical trials with *n* = 738 subjects with eGFR < 60 mL/min	3.3 mmHg BP reduction in placebo group
Goicoechea et al. (2010) [108]	Allopurinol vs. placebo 1:1	*n* = 113 with eGFR < 60 mL/min	Significant reduction of CRP level eGFR 1.3 mL/min/24 months increment
Golmohammadi et al. (2017) [109]	Allopurinol vs. placebo 1:1	*n* = 216 with eGFR 15–60 mL/min	Reduction of CKD decline with a mean difference of 1 mL/min/year
Bose et al. (2014, meta-analysis) [110]	Allopurinol vs. placebo 1:1	8 clinical trials with *n* = 476 with eGFR < 60 mL/min	Mean eGFR retarded by 3.3 mL/min/year in 5 from 8 studies
Lee et al. (2019) [111]	Allopurinol vs. Febuxostat vs placebo ½:½:1	*n* = 141, mean eGFR baseline = 42.1 mL/min	Febuxostat significant decreased UA level and maintain eGFR significant higher for 4 years in contrast to Allopurinol or placebo
Zhang et al. (2019) [112]	Febuxostat vs. Allopurinol 1:1	*n* = 152 with CKD stage 2–3	Febuxostat showed superiority in eGFR decline but not in proteinuria or uric acid control
Hsu et al. (2020, meta-analysis) [113]	Febuxostat vs. Allopurinol 1:1	*n* = 6057 with CKD stage 5	Lower risk of progression to dialysis on febuxostat group
Liu et al. (2018, meta-analysis) [115]	Uric-acid-lowering therapy	12 clinical trials with 832 CKD subjects	8 trials showed a mean serum creatinine reduction by −0.63
Sampson et al. (2017, meta-analysis) [116]	Uric-acid-lowering therapy	12 clinical trials with 1187 CKD subjects	Conflicting evidence—no apparent benefits in eGFR, blood pressure, or proteinuria control
Su et al. (2017, meta-analysis) [117]	Uric-acid-lowering therapy	16 clinical trial with 1211 CKD subjects	eGFR progression retarded by 4.1 mL/min/year 55% reduced risk of AKI 60% reduced risk in cardiovascular events
Chen et al. (2020, meta-analysis) [118]	Uric-acid-lowering therapy	28 trials with 6458 CKD subjects	No benefits in kidney failure or cardiovascular events
Lin et al. (2019, meta-analysis) [120]	Febuxostat vs. placebo	11 trials with 1317 CKD stage 3–4 subjects	Reno-protective effects with a mean difference in eGFR of 3.6 mL/min

HU, hyperuricemia; AKI, acute kidney injury; eGFR, estimated glomerular filtration; CRP, C-reactive protein; BP, blood pressure; UA, serum uric acid.

## Data Availability

Not applicable.

## References

[1] Adomako E., Moe O.W. (2020). Uric Acid and Urate in Urolithiasis: The Innocent Bystander, Instigator, and Perpetrator. Semin. Nephrol..

[2] Jakše B., Jakše B., Pajek M., Pajek J. (2019). Uric Acid and Plant-Based Nutrition. Nutrients.

[3] Caliceti C., Calabria D., Roda A., Cicero A. (2017). Fructose Intake, Serum Uric Acid, and Cardiometabolic Disorders: A Critical Review. Nutrients.

[4] Choi H.K., Mount D.B., Reginato A.M. (2005). Pathogenesis of gout. Ann. Intern. Med..

[5] Carbone A., Al Salhi Y., Tasca A., Palleschi G., Fuschi A., De Nunzio C., Bozzini G., Mazzaferro S., Pastore A.L. (2018). Obesity and kidney stone disease: A systematic review. Minerva Urol. Nefrol..

[6] Gaubert M., Bardin T., Cohen-Solal A., Diévart F., Fauvel J.P., Guieu R., Sadrin S., Maixent J.M., Galinier M., Paganelli F. (2020). Hyperuricemia and Hypertension, Coronary Artery Disease, Kidney Disease: From Concept to Practice. Int. J. Mol. Sci..

[7] Aktas G., Khalid A., Kurtkulagi O., Duman T.T., Bilgin S., Kahveci G., Atak Tel B.M., Sincer I., Gunes Y. (2022). Poorly controlled hypertension is associated with elevated serum uric acid to HDL-cholesterol ratio: A cross-sectional cohort study. Postgrad. Med..

[8] Zhu Y., Pandya B.J., Choi H.K. (2012). Comorbidities of gout and hyperuricemia in the US general population: NHANES 2007–2008. Am. J. Med..

[9] Novak S., Melkonian A.K., Patel P.A., Kleinman N.L., Joseph-Ridge N., Brook R.A. (2007). Metabolic syndrome-related conditions among people with and without gout: Prevalence and resource use. Curr. Med. Res. Opin..

[10] Heda R., Yazawa M., Shi M., Bhaskaran M., Aloor F.Z., Thuluvath P.J., Satapathy S.K. (2021). Non-alcoholic fatty liver and chronic kidney disease: Retrospect, introspect, and prospect. World J. Gastroenterol..

[11] Franco Á.O., Starosta R.T., Roriz-Cruz M. (2019). The specific impact of uremic toxins upon cognitive domains: A review. Braz. J. Nephrol..

[12] Park J.H., Jo Y.I., Lee J.H. (2020). Renal effects of uric acid: Hyperuricemia and hypouricemia. Korean J. Intern. Med..

[13] Shen C., Guo Y., Luo W., Lin C., Ding M. (2013). Serum urate and the risk of Parkinson’s disease: Results from a meta-analysis. Can. J. Neurol. Sci..

[14] Moccia M., Lanzillo R., Costabile T., Russo C., Carotenuto A., Sasso G., Postiglione E., De Luca Picione C., Vastola M., Maniscalco G.T. (2015). Uric acid in relapsing-remitting multiple sclerosis: A 2-year longitudinal study. J. Neurol..

[15] Bolayir A., Cigdem B., Gokce S.F., Yilmaz D. (2021). The relationship between neutrophil/lymphocyte ratio and uric acid levels in multiple sclerosis patients. Bratisl. Lek. Listy..

[16] Lu N., Dubreuil M., Zhang Y., Neogi T., Rai S.K., Ascherio A., Hernán M.A., Choi H.K. (2016). Gout and the risk of Alzheimer’s disease: A population-based, BMI-matched cohort study. Ann. Rheum. Dis..

[17] Roumeliotis S., Roumeliotis A., Dounousi E., Eleftheriadis T., Liakopoulos V. (2019). Dietary Antioxidant Supplements and Uric Acid in Chronic Kidney Disease: A Review. Nutrients.

[18] Keenan R.T. (2020). The biology of urate. Semin. Arthritis Rheum..

[19] Available online. https://pubchem.ncbi.nlm.nih.gov/compound/Uric-acid#section=Dissociation-Constants.

[20] El Ridi R., Tallima H. (2017). Physiological functions and pathogenic potential of uric acid: A review. J. Adv. Res..

[21] Jin M., Yang F., Yang I., Yin Y., Luo J.J., Wang H., Yang X.F. (2012). Uric acid, hyperuricemia and vascular diseases. Front. Biosci. Landmark Ed..

[22] Kimura Y., Tsukui D., Kono H. (2021). Uric Acid in Inflammation and the Pathogenesis of Atherosclerosis. Int. J. Mol. Sci..

[23] Waheed Y., Yang F., Sun D. (2021). Role of asymptomatic hyperuricemia in the progression of chronic kidney disease and cardiovascular disease. Korean J. Intern. Med..

[24] Kim K.M., Henderson G.N., Ouyang X., Frye R.F., Sautin Y.Y., Feig D.I., Johnson R.J. (2009). A sensitive and specific liquid chromatography-tandem mass spectrometry method for the determination of intracellular and extracellular uric acid. J. Chromatogr. B Analyt. Technol. Biomed. Life Sci..

[25] Su H.Y., Yang C., Liang D., Liu H.F. (2020). Research Advances in the Mechanisms of Hyperuricemia-Induced Renal Injury. Biomed. Res. Int..

[26] Miller S.G., Hafen P.S., Brault J.J. (2019). Increased Adenine Nucleotide Degradation in Skeletal Muscle Atrophy. Int. J. Mol. Sci..

[27] Petreski T., Ekart R., Hojs R., Bevc S. (2020). Hyperuricemia, the heart, and the kidneys—To treat or not to treat?. Ren. Fail..

[28] Nakagawa T., Sanchez-Lozada L.G., Andres-Hernando A., Kojima H., Kasahara M., Rodriguez-Iturbe B., Bjornstad P., Lanaspa M.A., Johnson R.J. (2021). Endogenous Fructose Metabolism Could Explain the Warburg Effect and the Protection of SGLT2 Inhibitors in Chronic Kidney Disease. Front. Immunol..

[29] Major T.J., Topless R.K., Dalbeth N., Merriman T.R. (2018). Evaluation of the diet wide contribution to serum urate levels: Meta-analysis of population based cohorts. BMJ.

[30] Mandal A.K., Mount D.B. (2015). The molecular physiology of uric acid homeostasis. Annu. Rev. Physiol..

[31] Wong K., Briddon S.J., Holliday N.D., Kerr I.D. (2016). Plasma membrane dynamics and tetrameric organisation of ABCG2 transporters in mammalian cells revealed by single particle imaging techniques. Biochim. Biophys. Acta.

[32] Mahaffey K.W., Li J., Badve S.V., Zhou Z., Oh R., Lee M., Perkovic V., de Zeeuw D., Fulcher G., Matthews D.R. (2019). The effects of canagliflozin on uric acid and gout in patients with type 2 diabetes in the CANVAS programme. Diabetologia.

[33] Roch-Ramel F., Guisan B. (1999). Renal transport of urate in humans. News Physiol. Sci..

[34] Nigam S.K., Bhatnagar V. (2018). The systems biology of uric acid transporters. Curr. Opin. Nephrol. Hypertens..

[35] Johnson R.J., Bakris G.L., Borghi C., Chonchol M.B., Feldman D., Lanaspa M.A., Merriman T.R., Moe O.W., Mount D.B., Sanchez Lozada L.G. (2018). Hyperuricemia, Acute and Chronic Kidney Disease, Hypertension, and Cardiovascular Disease: Report of a Scientific Workshop Organized by the National Kidney Foundation. Am. J. Kidney Dis..

[36] Fanelli G.M., Bohn D., Stafford S. (1970). Functional characteristics of renal urate transport in the Cebus monkey. Am. J. Physiol..

[37] Gershon S.L., Fox I.H. (1974). Pharmacologic effects of nicotinic acid on human purine metabolism. J. Lab. Clin. Med..

[38] Köttgen A., Albrecht E., Teumer A., Vitart V., Krumsiek J., Hundertmark C., Pistis G., Ruggiero D., O’Seaghdha C.M., Haller T. (2013). Genome-wide association analyses identify 18 new loci associated with serum urate concentrations. Nat. Genet..

[39] Goldfinger S., Klinenberg E., Seegmiller J.E. (1965). Renal retention of uric acid induced by infusion of beta-hydroxybutyrate and acetoacetate. N. Engl. J. Med..

[40] Uetake D., Ohno I., Ichida K., Yamaguchi Y., Saikawa H., Endou H., Hosoya T. (2010). Effect of fenofibrate on uric acid metabolism and urate transporter 1. Intern. Med..

[41] Nakashima M., Uematsu T., Kosuge K., Kanamaru M. (1992). Pilot study of the uricosuric effect of DuP-753, a new angiotensin II receptor antagonist, in healthy subjects. Eur. J. Clin. Pharmacol..

[42] Dinour D., Gray N.K., Campbell S., Shu X., Sawyer L., Richardson W., Rechavi G., Amariglio N., Ganon L., Sela B.A. (2010). Homozygous SLC2A9 mutations cause severe renal hypouricemia. J. Am. Soc. Nephrol..

[43] Woodward O.M., Köttgen A., Coresh J., Boerwinkle E., Guggino W.B., Köttgen M. (2009). Identification of a urate transporter, ABCG2, with a common functional polymorphism causing gout. Proc. Natl. Acad. Sci. USA.

[44] Woodward O.M., Tukaye D.N., Cui J., Greenwell P., Constantoulakis L.M., Parker B.S., Rao A., Köttgen M., Maloney P.C., Guggino W.B. (2013). Gout-causing Q141K mutation in ABCG2 leads to instability of the nucleotide-binding domain and can be corrected with small molecules. Proc. Natl. Acad. Sci. USA.

[45] Jutabha P., Anzai N., Kitamura K., Taniguchi A., Kaneko S., Yan K., Yamada H., Shimada H., Kimura T., Katada T. (2010). Human sodium phosphate transporter 4 (hNPT4/SLC17A3) as a common renal secretory pathway for drugs and urate. J. Biol. Chem..

[46] Maesaka J.K., Fishbane S. (1998). Regulation of renal urate excretion: A critical review. Am. J. Kidney Dis..

[47] Maiuolo J., Oppedisano F., Gratteri S., Muscoli C., Mollace V. (2016). Regulation of uric acid metabolism and excretion. Int. J. Cardiol..

[48] Egan B.M., Lackland D.T. (2000). Biochemical and Metabolic Effects of Very-Low-Salt Diets. Am. J. Med. Sci..

[49] Xin P., Jiang G.H., Zheng W.L., Fan L.L., Li C.K., Wang D.Z. (2021). Study on the diet balance index and its relationship with blood uric acid of smoking adults in Tianjin. Zhonghua Liu Xing Bing Xue Za Zhi.

[50] Quiñones Galvan A., Natali A., Baldi S., Frascerra S., Sanna G., Ciociaro D., Ferrannini E. (1995). Effect of insulin on uric acid excretion in humans. Am. J. Physiol..

[51] Flisiński M., Brymora A., Skoczylas-Makowska N., Stefańska A., Manitius J. (2021). Fructose-Rich Diet Is a Risk Factor for Metabolic Syndrome, Proximal Tubule Injury and Urolithiasis in Rats. Int. J. Mol. Sci..

[52] Chino Y., Kuwabara M., Hisatome I. (2021). Factors Influencing Change in Serum Uric Acid After Administration of the Sodium-Glucose Cotransporter 2 Inhibitor Luseogliflozin in Patients with Type 2 Diabetes Mellitus. J. Clin. Pharmacol..

[53] Moriwaki Y., Yamamoto T., Tsutsumi Z., Takahashi S., Hada T. (2002). Effects of angiotensin II infusion on renal excretion of purine bases and oxypurinol. Metabolism.

[54] Hui J.Y., Choi J.W.J., Mount D.B., Zhu Y., Zhang Y., Choi H.K. (2012). The independent association between parathyroid hormone levels and hyperuricemia: A national population study. Arthritis Res. Ther..

[55] Wu W., Dnyanmote A.V., Nigam S.K. (2011). Remote communication through solute carriers and ATP binding cassette drug transporter pathways: An update on the Remote Sensing and Signaling Hypothesis. Mol. Pharmacol..

[56] Zanoli L., Lentini P., Briet M., Castellino P., House A.A., London G.M., Malatino L., McCullough P.A., Mikhailidis D.P., Boutouyrie P. (2019). Arterial Stiffness in the Heart Disease of CKD. J. Am. Soc. Nephrol..

[57] Kang D.H., Park S.K., Lee I.K., Johnson R.J. (2005). Uric acid-induced C-reactive protein expression: Implication on cell proliferation and nitric oxide production of human vascular cells. J. Am. Soc. Nephrol..

[58] Zhou Y., You H., Zhang A., Jiang X., Pu Z., Xu G., Zhao M. (2020). Lipoxin A4 attenuates uric acid-activated, NADPH oxidase-dependent oxidative stress by interfering with translocation of p47phox in human umbilical vein endothelial cells. Exp. Ther. Med..

[59] Corry D.B., Eslami P., Yamamoto K., Nyby M.D., Makino H., Tuck M.L. (2008). Uric acid stimulates vascular smooth muscle cell proliferation and oxidative stress via the vascular renin-angiotensin system. J. Hyperten..

[60] Doğru S., Yaşar E., Yeşilkaya A. (2021). Uric acid can enhance MAPK pathway-mediated proliferation in rat primary vascular smooth muscle cells via controlling of mitochondria and caspase-dependent cell death. J. Recept. Signal Transduct. Res..

[61] Ruggiero C., Cherubini A., Miller E., Maggio M., Najjar S.S., Lauretani F., Bandinelli S., Senin U., Ferrucci L. (2007). Usefulness of Uric Acid to Predict Changes in C-Reactive Protein and Interleukin-6 in 3-Year Period in Italians Aged 21 to 98 Years. Am. J. Cardiol..

[62] Gruszka K., Drożdż T., Wojciechowska W., Jankowski P., Terlecki M., Bijak M., Hering D., Bilo G., Drożdż D., Rajzer M. (2022). Effects of uric acid-lowering therapy in patients with essential arterial hypertension. Blood Press. Monit..

[63] Ejaz A.A., Johnson R.J., Shimada M., Mohandas R., Alquadan K.F., Beaver T.M., Lapsia V., Dass B. (2019). The Role of Uric Acid in Acute Kidney Injury. Nephron.

[64] Isaka Y., Takabatake Y., Takahashi A., Saitoh T., Yoshimori T. (2016). Hyperuricemia-induced inflammasome and kidney diseases. Nephrol. Dial. Transplant..

[65] Shi Y., Chen W., Jalal D., Li Z., Chen W., Mao H., Yang Q., Johnson R.J., Yu X. (2012). Clinical outcome of hyperuricemia in IgA nephropathy: A retrospective cohort study and randomized controlled trial. Kidney Blood Press. Res..

[66] Joosten L.A.B., Crişan T.O., Bjornstad P., Johnson R.J. (2020). Asymptomatic hyperuricaemia: A silent activator of the innate immune system. Nat. Rev. Rheumatol..

[67] Miao Y., Ottenbros S.A., Laverman G.D., Brenner B.M., Cooper M.E., Parving H.H., Grobbee D.E., Shahinfar S., De Zeeuw D., Heerspink H.J.L. (2011). Effect of a reduction in uric acid on renal outcomes during losartan treatment: A post hoc analysis of the reduction of endpoints in non-insulin-dependent diabetes mellitus with the angiotensin ii antagonist losartan trial. Hypertension.

[68] Borghi C., Rosei E.A., Bardin T., Dawson J., Dominiczak A., Kielstein J.T., Manolis A.J., Perez-Ruiz F., Mancia G. (2015). Serum uric acid and the risk of cardiovascular and renal disease. J. Hypertens..

[69] Rapa S.F., Di Iorio B.R., Campiglia P., Heidland A., Marzocco S. (2020). Inflammation and oxidative stress in chronic kidney disease—potential therapeutic role of minerals, vitamins and plant-derived metabolites. Int. J. Mol. Sci..

[70] Hanna R.M., Ghobry L., Wassef O., Rhee C.M., Kalantar-Zadeh K. (2020). A Practical Approach to Nutrition, Protein-Energy Wasting, Sarcopenia, and Cachexia in Patients with Chronic Kidney Disease. Blood Purif..

[71] Grayson P.C., Young Kim S., Lavalley M., Choi H.K. (2011). Hyperuricemia and incident hypertension: A systematic review and meta-analysis. Arthritis Care Res..

[72] Wang J., Qin T., Chen J., Li Y., Wang L., Huang H., Li J. (2014). Hyperuricemia and risk of incident hypertension: A systematic review and meta-analysis of observational studies. PLoS ONE.

[73] Lv Q., Meng X.F., He F.F., Chen S., Su H., Xiong J., Gao P., Tian X.J., Liu J.S., Zhu Z.H. (2013). High Serum Uric Acid and Increased Risk of Type 2 Diabetes: A Systemic Review and Meta-Analysis of Prospective Cohort Studies. PLoS ONE.

[74] Kielstein J.T., Pontremoli R., Burnier M. (2020). Management of Hyperuricemia in Patients with Chronic Kidney Disease: A Focus on Renal Protection. Curr. Hypertens. Rep..

[75] Chonchol M., Shlipak M.G., Katz R., Sarnak M.J., Newman A.B., Siscovick D.S., Kestenbaum B., Carney J.K., Fried L.F. (2007). Relationship of uric acid with progression of kidney disease. Am. J. Kidney Dis..

[76] Luo Q., Xia X., Li B., Lin Z., Yu X., Huang F. (2019). Serum uric acid and cardiovascular mortality in chronic kidney disease: A meta-analysis. BMC Nephrol..

[77] Tsai C.W., Lin S.Y., Kuo C.C., Huang C.C. (2017). Serum Uric Acid and Progression of Kidney Disease: A Longitudinal Analysis and Mini-Review. PLoS ONE.

[78] Zhou Y., Zhao M., Pu Z., Xu G., Li X. (2018). Relationship between oxidative stress and inflammation in hyperuricemia Analysis based on asymptomatic young patients with primary hyperuricemia. Medicine.

[79] Yang T., Ding X., Wang Y., Zeng C., Wie J., Li H., Xiong Y.L., Gao S.G., Li Y.S., Lei G.H. (2016). Association between high-sensitivity C-reactive protein and hyperuricemia. Rheumatol. Int..

[80] Viazzi F., Piscitelli P., Giorda C., Ceriello A., Genovese S., Russo G., Guida P., Fioretto P., De Cosmo S., Pontremoli R. (2017). Metabolic syndrome, serum uric acid and renal risk in patients with T2D. PLoS ONE.

[81] Oikonen M., Wendelin-Saarenhovi M., Lyytikäinen L.P., Siitonen N., Loo B.M., Jula A., Seppälä I., Saarikoski L., Lehtimäki T., Hutri-Kähönen N. (2012). Associations between serum uric acid and markers of subclinical atherosclerosis in young adults. The cardiovascular risk in Young Finns study. Atherosclerosis.

[82] Palmer T.M., Nordestgaard B.G., Benn M., Tybjarg-Hansen A., Smith G.D., Lawlor D.A., Timpson N.J. (2013). Association of plasma uric acid with ischaemic heart disease and blood pressure: Mendelian randomization analysis of two large cohorts. BMJ.

[83] Sedaghat S., Pazoki R., Uitterlinden A.G., Hofman A., Stricker B.H.C., Ikram M.A., Franco O.H., Dehghan A. (2014). Association of uric acid genetic risk score with blood pressure: The rotterdam study. Hypertension.

[84] Mallamaci F., Testa A., Leonardis D., Tripepi R., Pisano A., Spoto B., Sanguedolce M.C., Parlongo R.M., Tripepi G., Zoccali C. (2014). A polymorphism in the major gene regulating serum uric acid associates with clinic SBP and the white-coat effect in a family-based study. J. Hypertens..

[85] Kleber M.E., Delgado G., Grammer T.B., Silbernagel G., Huang J., Krämer B.K., Ritz E., März W. (2015). Uric acid and cardiovascular events: A Mendelian randomization study. J. Am. Soc. Nephrol..

[86] Hughes K., Flynn T., De Zoysa J., Dalbeth N., Merriman T.R. (2014). Mendelian randomization analysis associates increased serum urate, due to genetic variation in uric acid transporters, with improved renal function. Kidney Int..

[87] Niu S.W., Hung C.C., Lin H.Y., Kuo I.C., Huang J.C., He J.S., Wen Z.H., Liang P.I., Chiu Y.W., Chang J.M. (2022). Reduced Incidence of Stroke in Patients with Gout Using Benzbromarone. J. Pers. Med..

[88] Rana P., Aleo M.D., Wen X., Kogut S. (2021). Hepatotoxicity reports in the FDA adverse event reporting system database: A comparison of drugs that cause injury via mitochondrial or other mechanisms. Acta Pharm. Sin. B.

[89] Kuriyama S. (2020). Dotinurad: A novel selective urate reabsorption inhibitor as a future therapeutic option for hyperuricemia. Clin. Exp. Nephrol..

[90] Bove M., Cicero A.F.G., Veronesi M., Borghi C. (2017). An evidence-based review on urate-lowering treatments: Implications for optimal treatment of chronic hyperuricemia. Vasc. Health Risk Manag..

[91] Ejaz A.A., Nakagawa T., Kanbay M., Kuwabara M., Kumar A., Garcia Arroyo F.E., Roncal-Jimenez C., Sasai F., Kang D.H., Jensen T. (2020). Hyperuricemia in Kidney Disease: A Major Risk Factor for Cardiovascular Events, Vascular Calcification, and Renal Damage. Semin. Nephrol..

[92] Bailey C.J. (2019). Uric acid and the cardio-renal effects of SGLT2 inhibitors. Diabetes Obes. Metab..

[93] Takata T., Isomoto H. (2021). Pleiotropic Effects of Sodium-Glucose Cotransporter-2 Inhibitors: Renoprotective Mechanisms beyond Glycemic Control. Int. J. Mol. Sci..

[94] Dekkers C.C.J., Gansevoort R.T., Heerspink H.J.L. (2018). New Diabetes Therapies and Diabetic Kidney Disease Progression: The Role of SGLT-2 Inhibitors. Curr. Diab. Rep..

[95] Dekkers C.C.J., Petrykiv S., Laverman G.D., Cherney D.Z., Gansevoort R.T., Heerspink H.J.L. (2018). Effects of the SGLT-2 inhibitor dapagliflozin on glomerular and tubular injury markers. Diabetes Obes. Metab..

[96] Baer P.C., Koch B., Freitag J., Schubert R., Geiger H. (2020). No Cytotoxic and Inflammatory Effects of Empagliflozin and Dapagliflozin on Primary Renal Proximal Tubular Epithelial Cells under Diabetic Conditions In Vitro. Int. J. Mol. Sci..

[97] Shaffner J., Chen B., Malhotra D.K., Dworkin L.D., Gong R. (2021). Therapeutic Targeting of SGLT2: A New Era in the Treatment of Diabetes and Diabetic Kidney Disease. Front. Endocrinol..

[98] Van Bommel E.J.M., Muskiet M.H.A., Tonneijck L., Kramer M.H.H., Nieuwdorp M., van Raalte D.H. (2017). SGLT2 Inhibition in the Diabetic Kidney—From Mechanisms to Clinical Outcome. Clin. J. Am. Soc. Nephrol..

[99] Hu J., Xu W., Yang H., Mu L. (2021). Uric acid participating in female reproductive disorders: A review. Reprod. Biol. Endocrinol..

[100] Giandalia A., Giuffrida A.E., Gembillo G., Cucinotta D., Squadrito G., Santoro D., Russo G.T. (2021). Gender differences in diabetic kidney disease: Focus on hormonal, genetic and clinical factors. Int. J. Mol. Sci..

[101] Limirio L.S., Santos H.O., Dos Reis A.S., de Oliveira E.P. (2021). Association Between Dietary Intake and Serum Uric Acid Levels in Kidney Transplant Patients. J. Ren. Nutr..

[102] Ma J., Han R., Cui T., Yang C., Wang S. (2022). Effects of high serum uric acid levels on oxidative stress levels and semen parameters in male infertile patients. Medicine.

[103] Choi H.K., Curhan G. (2007). Coffee, tea, and caffeine consumption and serum uric acid level: The third national health and nutrition examination survey. Arthritis Rheum..

[104] Guthikonda S., Sinkey C., Barenz T., Haynes W.G. (2003). Xanthine oxidase inhibition reverses endothelial dysfunction in heavy smokers. Circulation.

[105] Furuhashi M. (2020). New insights into purine metabolism in metabolic diseases: Role of xanthine oxidoreductase activity. Am. J. Physiol. Endocrinol. Metab..

[106] Bove M., Cicero A.F.G., Borghi C. (2017). The Effect of Xanthine Oxidase Inhibitors on Blood Pressure and Renal Function. Curr. Hypertens. Rep..

[107] Agarwal V., Hans N., Messerli F.H. (2013). Effect of Allopurinol on Blood Pressure: A Systematic Review and Meta-Analysis. J. Clin. Hypertens..

[108] Goicoechea M., de Vinuesa S.G., Verdalles U., Ruiz-Caro C., Ampuero J., Rincón A., Arroyo D., Luño J. (2010). Effect of allopurinol in chronic kidney disease progression and cardiovascular risk. Clin. J. Am. Soc. Nephrol..

[109] Golmohammadi S., Almasi A., Manouchehri M., Omrani H.R., Zandkarimi M.R. (2017). Allopurinol against Progression of Chronic Kidney Disease. Iran. J. Kidney Dis..

[110] Bose B., Badve S.V., Hiremath S.S., Boudville N., Brown F.G., Cass A., de Zoysa J.R., Fassett R.G., Faull R., Harris D.C. (2014). Effects of uric acid-lowering therapy on renal outcomes: A systematic review and meta-analysis. Nephrol. Dial. Transplant..

[111] Lee J.W., Lee K.H. (2019). Comparison of renoprotective effects of febuxostat and allopurinol in hyperuricemic patients with chronic kidney disease. Int. Urol. Nephrol..

[112] Zhang X., Wan D., Yang G., Peng Q., Wang X. (2019). Febuxostat is superior to allopurinol in delaying the progression of renal impairment in patients with chronic kidney disease and hyperuricemia. Int. Urol. Nephrol..

[113] Hsu Y.O., Wu I.W., Chang S.H., Lee C.C., Tsai C.Y., Lin C.Y., Lin W.T., Huang Y.T., Wu C.Y., Kuo G. (2020). Comparative Renoprotective Effect of Febuxostat and Allopurinol in Predialysis Stage 5 Chronic Kidney Disease Patients: A Nationwide Database Analysis. Clin. Pharmacol. Ther..

[114] Mauer M., Doria A. (2020). Uric acid and risk of diabetic kidney disease. J. Nephrol..

[115] Liu X., Zhai T., Ma R., Luo C., Wang H., Liu L. (2018). Effects of uric acid-lowering therapy on the progression of chronic kidney disease: A systematic review and meta-analysis. Ren. Fail..

[116] Sampson A.L., Singer R.F., Walters G.D. (2017). Uric acid lowering therapies for preventing or delaying the progression of chronic kidney disease. Cochrane Database Syst. Rev..

[117] Su X., Xu B., Yan B., Qiao X., Wang L. (2017). Effects of uric acid-lowering therapy in patients with chronic kidney disease: A meta-analysis. PLoS ONE.

[118] Chen Q., Wang Z., Zhou J., Chen Z., Li Y., Li S., Zhao H., Badve S.V., Lv J. (2020). Effect of Urate-Lowering Therapy on Cardiovascular and Kidney Outcomes: A Systematic Review and Meta-Analysis. Clin. J. Am. Soc. Nephrol..

[119] Li X., Meng X., Timofeeva M., Tzoulaki I., Tsilidis K.K., Ioannidis J.P., Campbell H., Theodoratou E. (2017). Serum uric acid levels and multiple health outcomes: Umbrella review of evidence from observational studies, randomised controlled trials, and Mendelian randomisation studies. BMJ.

[120] Lin T.C., Hung L.Y., Chen Y.C., Lo W.C., Lin C.H., Tam K.W., Wu M.Y. (2019). Effects of febuxostat on renal function in patients with chronic kidney disease: A systematic review and meta-analysis. Medicine.

[121] FitzGerald J.D., Dalbeth N., Mikuls T., Brignardello-Petersen R., Guyatt G., Abeles A.M., Gelber A.C., Harrold L.R., Khanna D., King C. (2020). 2020 American College of Rheumatology Guideline for the Management of Gout. Arthritis Care Res..

[122] Yamanaka H. (2011). Japanese Society of Gout and Nucleic Acid Metabolism. Japanese guideline for the management of hyperuricemia and gout: Second edition. Nucleosides Nucleotides Nucleic Acids.

[123] Singh J.A., Edwards N.L. (2018). EULAR gout treatment guidelines by Richette et al.: Uric acid and neurocognition. Ann. Rheum. Dis..

[124] Vanholder R., Pletinck A., Schepers E., Glorieux G. (2018). Biochemical and Clinical Impact of Organic Uremic Retention Solutes: A Comprehensive Update. Toxins.

[125] Viggiano D., Gigliotti G., Vallone G., Giammarino A., Nigro M., Capasso G. (2018). Urate-Lowering Agents in Asymptomatic Hyperuricemia: Role of Urine Sediment Analysis and Musculoskeletal Ultrasound. Kidney Blood Press. Res..

